# Mycophenolate Mofetil, an Inhibitor of Inosine Monophosphate Dehydrogenase, and Tofacitinib, a Janus Kinase Inhibitor, Attenuate Airway Inflammation and Hyperresponsiveness in a Mouse Model of Allergic Asthma

**DOI:** 10.3390/molecules29225293

**Published:** 2024-11-09

**Authors:** Bernard Kravčenia, Tomasz Maślanka

**Affiliations:** Department of Pharmacology and Toxicology, Faculty of Veterinary Medicine, University of Warmia and Mazury in Olsztyn, Oczapowskiego Street 13, 10-719 Olsztyn, Poland; bernard.kravcenia@student.uwm.edu.pl

**Keywords:** asthma, anti-asthmatic drugs, mycophenolate mofetil, tofacitinib, JAK/STAT pathway, CD4^+^ T cells, Treg cells

## Abstract

Treatment-resistant asthma remains an unresolved clinical problem and a challenge for current medical science. Consequently, there is a growing and urgent need to develop novel or alternative therapeutic options for the treatment of asthma. The research problem raised in this study was to assess and compare mycophenolate mofetil (MMF), an inhibitor of inosine monophosphate dehydrogenase, and tofacitinib (TFB), a Janus kinase inhibitor, for anti-asthmatic properties, and consequently to determine whether these agents may have potential as alternative options for treatment of allergic asthma. For this purpose, we assessed the effect of administration of MMF and TFB on the development of a mouse model of allergic airway inflammation (AAI) and accompanying CD4^+^ (cluster of differentiation 4) T-cell immune response in the lung-draining mediastinal lymph nodes (MLNs) and lungs, i.e., in the inductive and effector sites, respectively, of the immune response underlying the development of allergic asthma. The results from a histopathological scoring system demonstrated that the administration of MMF and TFB did not prevent or abolish ovalbumin-induced AAI, but strongly attenuated its severity. The pulmonary function tests revealed that the treatment with MMF and TFB significantly reduced methacholine-induced bronchoconstriction. These results indicate that the treatment with TFB and MMF attenuated the development of ovalbumin-induced AAI. The magnitude of the anti-asthmatic effect was comparable between both agents. The study revealed that the impairment of the clonal expansion of effector CD4^+^ T cells in the MLNs is a critical event in the mechanism underlying the anti-asthmatic effect of MMF and TFB. Apart from this, the findings of the study strongly suggest that the suppression of the interleukin-33/suppression of tumorigenicity-2 signaling pathway may constitute an additional mechanism responsible for producing this effect. In turn, the results indicate that the anti-asthmatic action induced by the studied agents is not mediated by the generation of forkhead box protein 3-expressing CD4^+^ regulatory T cells. Clinical implication of the results: the results suggest that MMF and TFB may exert anti-asthmatic action, and thus they may be considered therapeutic options for the treatment of allergic asthma cases resistant to conventional/existing treatment.

## 1. Introduction

Asthma is the most prevalent chronic respiratory disease worldwide, affecting more than 300 million people of all ethnic groups and all ages [1]. Allergic asthma is the most common type of the disease (60% of all cases) [2]. It is a chronic inflammatory disease characterized by inflammation in and around the airways, mucus hypersecretion, reversible airway hyperresponsiveness (AHR) and fibrosis, resulting in obstruction and remodeling of the airways [3]. Inhaled, and sometimes systemic, steroidal anti-inflammatory drugs, more often referred to as glucocorticosteroids (GCs), are the mainstay of asthma therapy owing to their complex and multidirectional mechanism of action, including the suppression of multiple inflammatory genes [4]; in additional to the standard therapy, other treatments can be applied, for instance bacterial lysates can benefit patients with asthma [5]. However, the long-term use of inhaled GCs may cause side effects that are found with much higher doses of systemic GCs [6]. Perhaps even more importantly, about 10% of asthmatics are refractory to GC therapy, and these patients are referred to as steroid-resistant asthmatics [7,8].

**Figure 1:**
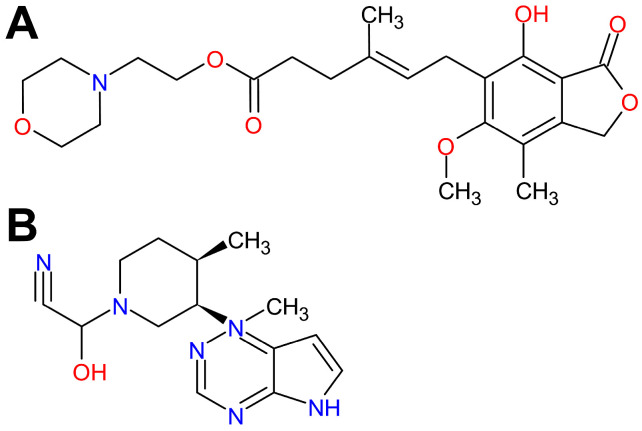
Chemical structure of mycophenolate mofetil (**A**) and tofacitinib (**B**).

Treatment-resistant asthma remains an unresolved clinical problem and a challenge for current medical science. Consequently, there is a growing and urgent need to develop novel or alternative therapeutic strategies/options for the treatment of asthma, especially allergic asthma. Therefore, researchers are constantly looking for new therapeutic targets in allergic asthma or, alternatively, they are investigating existing immunosuppressive and/or anti-inflammatory drugs for anti-asthmatic properties. The present study inscribes itself in this scientific trend. The research problem raised in this study was to assess and compare mycophenolate mofetil (MMF), an inhibitor of inosine monophosphate dehydrogenase (IMPDH), and tofacitinib (TFB), a Janus kinase (JAK) inhibitor (Figure 1), for anti-asthmatic properties, and consequently to determine whether these agents may have potential as an alternative option for treatment of allergic asthma. For this purpose, we assessed the effect of administration of MMF and TFB on the development of a mouse model of allergic airway inflammation (AAI) and accompanying CD4^+^ (cluster of differentiation 4) T-cell immune response in the mediastinal lymph nodes (MLNs) and lungs, i.e., in the inductive and effector sites, respectively, of the immune response underlying the development of allergic asthma.

Mycophenolate mofetil (MMF), an inactive prodrug of mycophenolic acid (MPA), is an immunosuppressive drug that was initially introduced for the treatment of the graft-versus-host disease in organ transplantation [9,10]. However, over the last 30 years, the indications for the drug have been extended to the treatment of many autoimmune diseases. MMF is currently used for the treatment of lupus nephritis, interstitial lung disease associated with systemic sclerosis, dermatomyositis, and systemic vasculitis while being efficacious also as rescue therapy in various orphan diseases [11]. The active metabolite of MMF, MPA, is a selective, non-competitive, and reversible inhibitor of inosine monophosphate dehydrogenase (IMPDH), a key enzyme in the de novo synthesis of guanine nucleotides, which is preferentially expressed in activated lymphocytes [12]. In this way, MPA induces inhibition of T and B lymphocyte proliferation and maturation, thereby causing the suppression of cellular and humoral immune responses [9,10]. Considering the above, we hypothesized that MMF would prevent or attenuate the development of AAI through inhibition of clonal expansion of CD4^+^ effector T (Teff) cells in the lung-draining MLNs, which in consequence would prevent or reduce the infiltration of inflammatory cells into the lung tissue.

The JAK-signal transducer and activator of transcription (STAT) signaling pathway, including 4 JAK intracellular tyrosine kinases (i.e., JAK1, JAK2, JAK3, and TYK2), represents a crucial system involved in the transduction of signaling of several cytokines related to immune responses, cell activation, survival, inflammation, and autoimmunity [13,14]. JAKs activate STAT proteins, which translocate to the nucleus and modulate the expression of their downstream target genes, including those involved in the development of inflammation, e.g., those coding for pro-inflammatory cytokines [14,15]. The JAK/STAT pathway may be considered a promising target for asthma treatment because most of the inflammatory pathways implicated in different asthma endotypes involve cytokines and growth factors that signal via receptors coupled to JAKs asthma [15]. Moreover, studies with genetically modified mouse models revealed an essential role of the JAK/STAT pathway in immune-mediated diseases, including asthma [15]. Therefore, the inhibition of the JAK-STAT signaling pathway using JAK inhibitors may represent an attractive therapeutic approach for asthma treatment. In fact, there are reports in the available literature describing the effect of different JAK inhibitors on selected parameters evaluating the development of animal models of lung inflammation [16,17,18,19,20]. The results of these experimental studies as well as the results of several clinical trials [21] indicate that JAK inhibitors have high therapeutic potential in asthma [15].

TFB, a selective Janus kinase (JAK) 1 and 3 inhibitor, was the second JAK inhibitor approved by the FDA (2012) for use in a clinical setting. The drug was approved originally for the treatment of rheumatoid arthritis. Over the following years, the indications for TFB have expanded significantly, with FDA approvals for treating psoriatic arthritis, ulcerative colitis, alopecia areata and polyarticular course juvenile idiopathic arthritis [22]. The pharmaceutical research has shown successful delivery of TFB via inhalation [23]. In our opinion, TFB may have special potential as an alternative option for treatment of steroid-resistant allergic asthma. This is justified by the following arguments: (a) TFB is approved in many countries; (b) for over ten years of therapeutical use now, TFB has appeared to be effective and relatively safe; (c) it is possible to develop an inhalation formulation of the drug; (d) the expanding indications for TFB use strongly suggest its universal efficacy in treatment of T cell-mediated diseases. It is worth emphasizing that the mechanism of action of TFB, as well as other JAK inhibitors, is completely different from that of glucocorticoids, hence these agents may be particularly effective in patients with refractory steroid-resistant asthma. Based on the above rationale, it has been deemed justifiable and essential to assess TFB in terms of its potential use as anti-asthmatic agent.

Although there are reports in the available literature on the effect of different JAK inhibitors on the development of animal models of lung inflammation [16,17,18,19,20], their effect on the accompanying CD4^+^ T cell immune response in the inductive and effector compartments associated with pathogenesis of allergic asthma has not been investigated thus far. Therefore, another goal of this study was to expand the knowledge and understanding of the mechanism underlying the anti-asthmatic properties of JAK inhibitors and the involvement of forkhead box protein 3 (Foxp3)-expressing CD4^+^ Teff and regulatory (Treg) T cells in this mechanism.

## 2. Results

### 2.1. Interpretation of the Results

#### 2.1.1. Ovalbumin(OVA)-Induced Effect

OVA immunization led to a significant increase in the value of the parameter in vehicle-treated mice (OVA + VEH group) compared to vehicle-treated non-immunized mice (PBS + VEH group).

#### 2.1.2. Prevention/Abolishment of OVA-Induced Effect

The value of the parameter was significantly lower in MMF- and/or TFB-treated OVA-immunized mice than in the OVA + VEH group, and did not differ significantly from the value of this parameter achieved in the PBS + VEH group.

#### 2.1.3. Reduction/Attenuation of OVA-Induced Effect

The value of the parameter was significantly lower in MMF- and/or TFB-treated OVA-immunized mice than in the OVA + VEH group, but still higher than that in the PBS + VEH group.

#### 2.1.4. Decrease in the Value of Parameter

The value of the parameter was significantly lower in MMF- and/or TFB-treated OVA-immunized mice than in the PBS + VEH group.

### 2.2. MMF and TFB Do Not Prevent, but Attenuate OVA-Induced AAI

**Figure 2:**
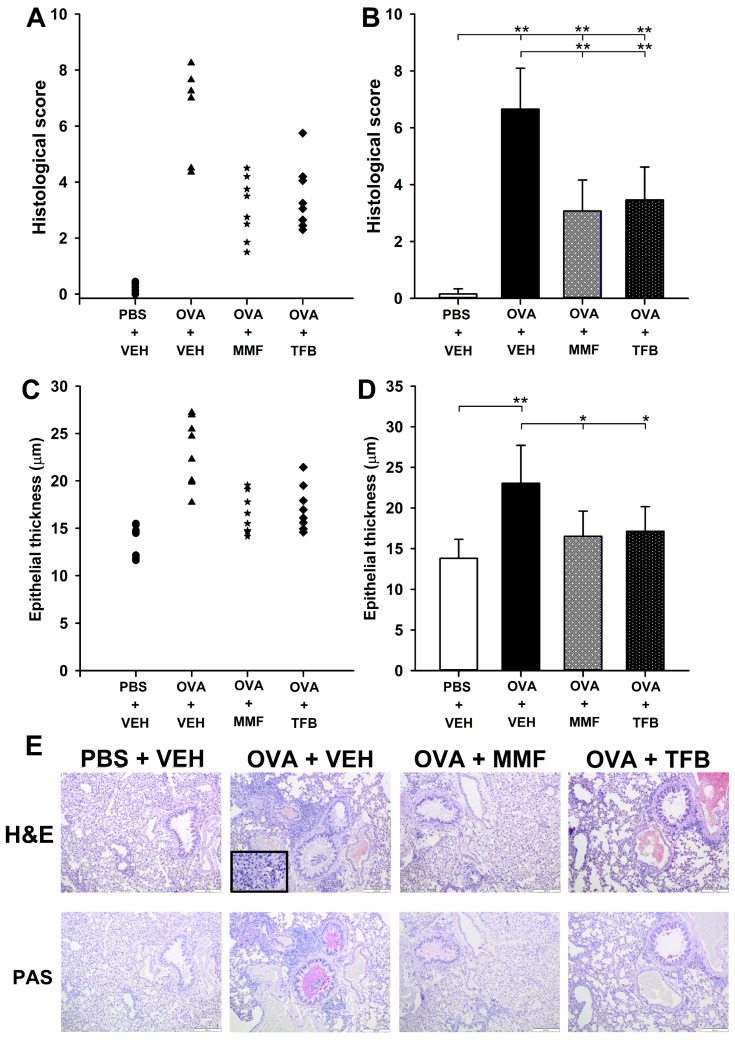
Effect of mycophenolate mofetil (MMF) and tofacitinib (TFB) on allergic airway inflammation (AAI) in ovalbumin (OVA)-immunized mice. The histological scores of airway inflammation and airway epithelial thickness are expressed for individual mice ((**A**,**C**); the symbols represent individual mice) and as a group mean ((**B**,**D**); the bars depict group mean). The mean (±S.D.) results from two independent experiments with four mice per group are shown (overall *n* = 8 per group, * *p* < 0.01, ** *p* < 0.001). Examples of photomicrographs of tissue sections stained with hematoxylin and eosin (H&E) and periodic acid-Schiff (PAS) (**E**). Densely infiltrated eosinophils and lymphocytes (enlargement of the rectangle in the upper photograph) with mucous hypersecretion are visible in lung sections of VEH-treated, OVA-immunized mouse, while there are no histopathological abnormalities in sections of VEH-treated, non-immunized mouse. Mild AAI can be seen in OVA-immunized mice treated with MMF and TFB (**E**). Magnification: 10-fold; 100-fold for the insert.

All mice in the OVA + VEH group developed allergic inflammation typical of OVA-induced AAI, including airway epithelium thickening and infiltration of inflammatory cells around the bronchi and bronchioles. The infiltrates were composed of eosinophils, neutrophils and lymphocytes (Figure 2E, H&E). Goblet cell hyperplasia and mucus hypersecretion were observed (Figure 2E, PAS). In contrast, there were no features of airway inflammation in PBS + VEH group (Figure 2E).

The scoring system was used for quantification of histopathological changes in the lungs. Immunization with OVA induced a significant increase in the mean scores of lung histologic lesions (Figure 2B). Six mice in the OVA + VEH group developed severe AAI (the histological score of these mice ranged within 7.0–8.25), while two mice developed moderate AAI (the histological score of these mice: 4.35–4.50) (Figure 2A). The mean score in the OVA + VEH group (6.66 ± 1.44) was over 44-fold higher than that in the PBS + VEH group (0.15 ± 0.19). The mean score in the OVA + MMF and OVA + TFB groups was significantly higher compared to that in the PBS + VEH group, but lower than in the OVA + VEH group (Figure 2B). However, the comparison of the histological scores obtained for individual mice in the OVA + MMF and OVA + TFB groups clearly indicates that some mice developed minimal or very mild AAI, while others developed moderate AAI (Figure 2A); one mouse in OVA + TFB developed a more severe form of AAI (Figure 2A). The statistical analysis did not reveal any significant differences between the mean score in the OVA + MMF group and that in the OVA + TFB group (Figure 2B,D).

Quantitative assessment of the thickness of bronchial epithelium demonstrated that the individual values of this parameter were greater in all mice from the OVA + VEH group compared to those in mice from the PBS + VEH group (Figure 2C). The mean thickness of bronchial epithelium was significantly increased in the OVA + VEH group compared to the PBS + VEH group (Figure 2C,D). The mean thickness of epithelium in the OVA + MMF and OVA + TFB groups was considerably smaller than the value of this parameter in the OVA + VEH group and did not differ from that in the PBS + VEH group (Figure 2D). Thus, these results indicate that the administration of MMF and TFB prevented the OVA-induced increase in the thickness of bronchial epithelium. However, considering the overall histopathological results, it should be concluded that the treatment with MMF and TFB considerably attenuated, but did not prevent, OVA-induced AAI and this beneficial effect of the agents may differ between individuals.

### 2.3. MMF Reduces OVA-Induced Increases in the Values of Enhanced Pause (Penh) and Pause (PAU), While TFB Prevents and Reduces, Respectively, These Increases

**Figure 3:**
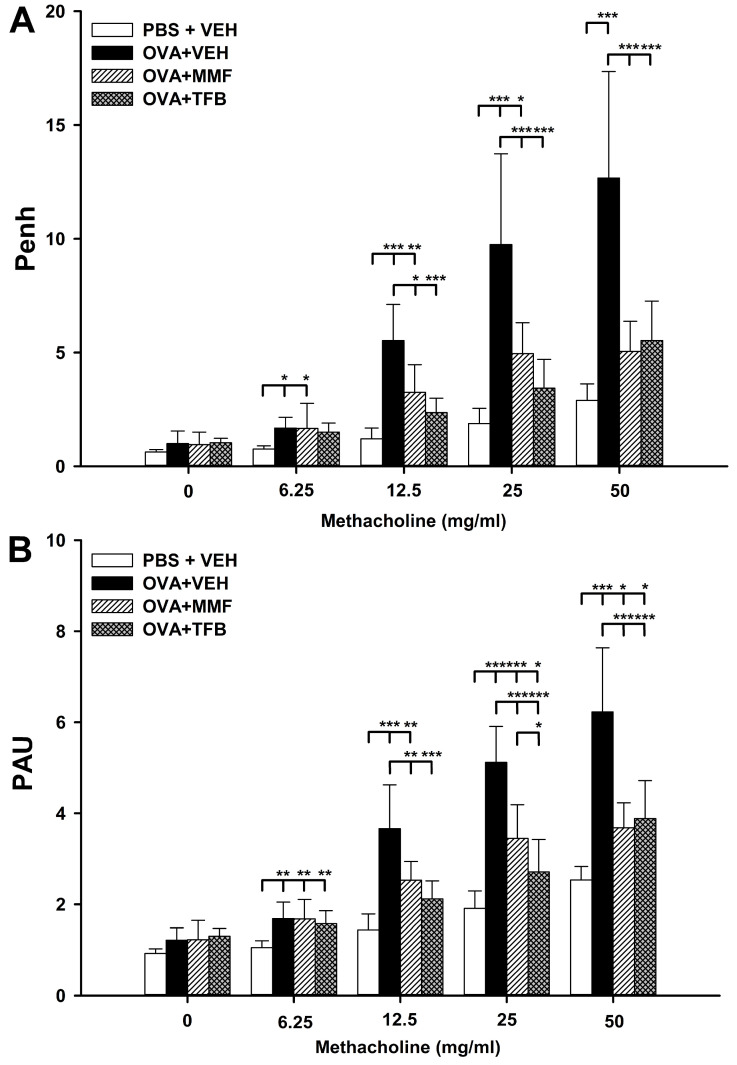
Effect of mycophenolate mofetil (MMF) and tofacitinib (TFB) on airway hyperresponsiveness (AHR) to inhaled methacholine (MCh).The AHR was measured as enhanced pause (Penh; (**A**)) and pause (PAU; (**B**)) 24 h after the last OVA challenge; both parameters are indicators of bronchoconstriction) in response to a nebulized challenge with MCh. Penh and PAU are expressed as the mean (±S.D.) from two independent experiments with four mice per group (overall *n* = 8 per group, * *p* < 0.05, ** *p* < 0.01, *** *p* < 0.001).

Along with the increasing concentration of methacholine (MCh), a relatively proportional increase occurred in the values of both assessed respiratory parameters in the OVA + VEH group. The mean Penh values at concentrations of 0, 6.25, 12.5, 25 and 50 mg/mL of MCh were 5.13, 3.30, 3.32, 5.95 and 6.46 times higher, respectively, than those at corresponding concentrations of MCh in the PBS + VEH group (Figure 3A). Similarly, the mean PAU values at concentrations of 0, 6.25, 12.5, 25 and 50 mg/mL of MCh were 2.70, 2.40, 2.80, 2.10 and 4.75 times higher, respectively, than those at corresponding concentrations of MCh in the PBS + VEH group (Figure 3B). These results indicate that the allergic asthma model was successfully induced.

The mean Penh values in the OVA + MMF group at concentrations of 6.25, 12.5 and 25 mg/mL of MCh were significantly higher compared to those in the PBS + VEH group, while there was no difference between those groups in this respect at the highest concentration of MCh (Figure 3A). In turn, the mean Penh values in the OVA + MMF group at concentrations 12.5, 25 and 50 mg/mL of MCh were significantly lower than those in the OVA + VEH group (Figure 3A). These results indicate that the treatment with MMF significantly reduced, but did not prevent, the OVA-induced increase in Penh values.

At none of the concentrations of MCh was there a significant difference in the mean Penh values between the PBS + VEH and OVA + TFB groups (Figure 3A). In turn, the mean Penh values in the OVA + TFB group at concentrations of 12.5, 25 and 50 mg/mL of MCh were significantly lower than those in the OVA + VEH group (Figure 3A). In the context of statistical analysis, the results indicate that treatment with TFB abolished the OVA-induced increase in Penh values. However, despite the lack of essential statistical differences between the PBS + VEH and OVA + TFB groups, the mean Penh values at concentrations of 6.25, 12.5, 25 and 50 mg/mL of MCh were 2.70, 1.30, 1.90 and 2.40 times higher, respectively, in the OVA + TFB group than those at corresponding concentrations of MCh in the PBS + VEH group (Figure 3A). Therefore, for reasons of scientific caution, the authors suggest that it would be more appropriate to conclude that the administration of TFB almost, but not fully, prevented the OVA-induced increase in Penh values. The statistical analysis did not reveal any crucial differences between the OVA + MMF and OVA + TFB groups (Figure 3A).

The mean PAU values in the OVA + MMF and OVA + TFB groups were significantly higher compared to those in the PBS + VEH group at almost all concentrations of MCh (with the exception of concentration 12.5 mg/mL in the OVA + TFB group) (Figure 3B). In turn, the mean PAU values in both treatment groups at concentrations 12.5, 25 and 50 mg/mL of MCh were significantly lower than those in the OVA + VEH group (Figure 3B). Thus, the study demonstrated that the administration of MMF and TFB significantly reduced, but did not prevent, an OVA-induced increase in PAU values. The mean PAU value in the OVA + TFB group at a concentration of 25 mg/mL of MCh was lower compared to that in the OVA + MMF group; the statistical analysis did not reveal any significant differences between these groups at remaining concentrations of MCh (Figure 3B).

### 2.4. MMF and TFB Prevents OVA-Induced Increase in the Count of Non-Treg CD4^+^ T Cells in the Lungs

**Figure 4:**
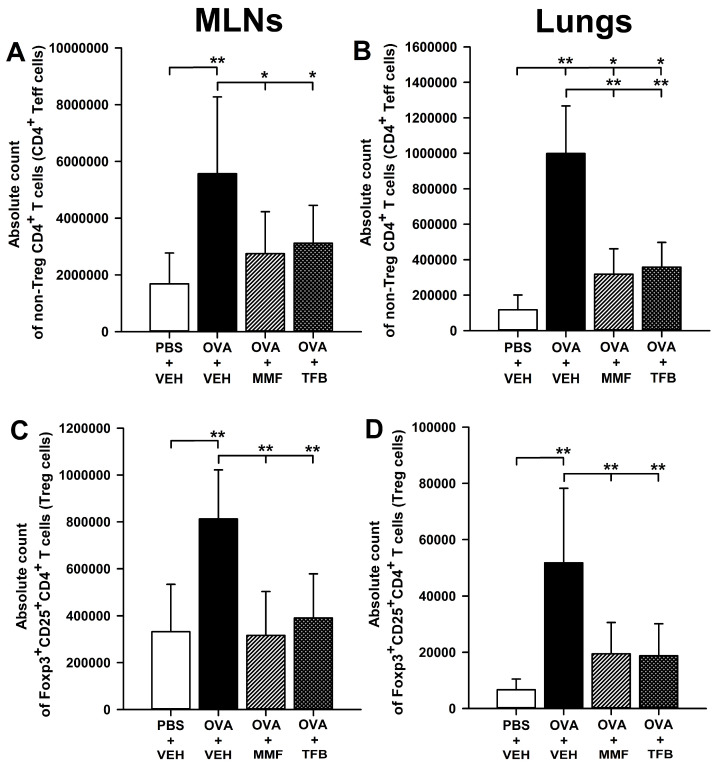
Effect of mycophenolate mofetil (MMF) and tofacitinib (TFB) on the number of regulatory (Treg) and non-Treg CD4^+^ T cells in the mediastinal lymph nodes (MLNs) and lungs. The absolute counts represent the number of non-Foxp3^+^CD25^+^ (non-Treg; (**A**,**B**)) and Foxp3^+^CD25^+^ (Treg; (**C**,**D**)) CD4^+^ T cells in the whole MLNs (**A**,**C**) and lungs (**B**,**D**) collected from individual mice. Results are expressed as the mean (±S.D.) of two independent experiments with four mice per group (overall *n* = 8 per group, * *p* < 0.05, ** *p* < 0.001).

The research showed that OVA immunization led to a significant increase in the absolute number of non-Foxp3^+^CD25^+^ (non-Treg; Figure 4A,B) and Foxp3^+^CD25^+^ (Treg; Figure 4C,D) CD4^+^ T cells in the MLNs (Figure 4A,C) and lungs (Figure 4B,D). The absolute number of non-Treg and Treg cells in the MLNs of mice treated with MMF and TFB did not differ significantly from the values of this parameter obtained in the PBS + VEH group, while being considerably lower than in the OVA + VEH group (Figure 4A,C). The same results were also obtained for Treg cells in the lungs (Figure 4D). Thus, the results indicate that the OVA-induced increase in the absolute number of non-Treg cells in the MLNs and Treg CD4^+^ T cells in the MLNs and lungs was prevented by the treatment with MMF and TFB (Figure 4A,C,D). However, despite the lack of essential statistical differences in the absolute number of Treg cells in the lungs between the PBS + VEH group and OVA + MMF and OVA + TFB groups, a trend toward increasing this parameter can be noticed in both treatment groups: the absolute number of Treg cells in the lungs of mice treated with MMF and TFB was 2.92 and 2.82 times greater, respectively, than that in mice from the PBS + VEH group. It seems that there was also a trend toward slightly increasing the absolute number of non-Treg CD4^+^ T cells in the MLNs of mice from both treatment groups: the absolute number of these cells in the OVA + MMF and OVA + TFB groups was 1.63 and 1.85 times greater, respectively, than the value of this parameter in the PBS + VEH group.

The absolute number of non-Treg cells in the lungs of mice from the OVA + MMF and OVA + TFB groups was significantly higher compared to the value of this parameter in the PBS + VEH group, but lower than that in the OVA + VEH group (Figure 4B). No differences were found in the value of this parameter between both treatment groups (Figure 4B). Thus, the study demonstrated that the treatment with MMF and TFB did not prevent, but reduced to a comparable degree, the OVA-induced increase in the absolute number of non-Treg CD4^+^ T cells in the lungs.

### 2.5. MMF and TFB Decrease the Percentage of Suppression of Tumorigenicity-2(ST2)^+^ Cells Within the CD4^+^ T Cell Subset in the MLNs and Prevent OVA-Induced Increase in the Absolute Number of ST2^+^CD4^+^ T Cells in the MLNs and Lungs

**Figure 5:**
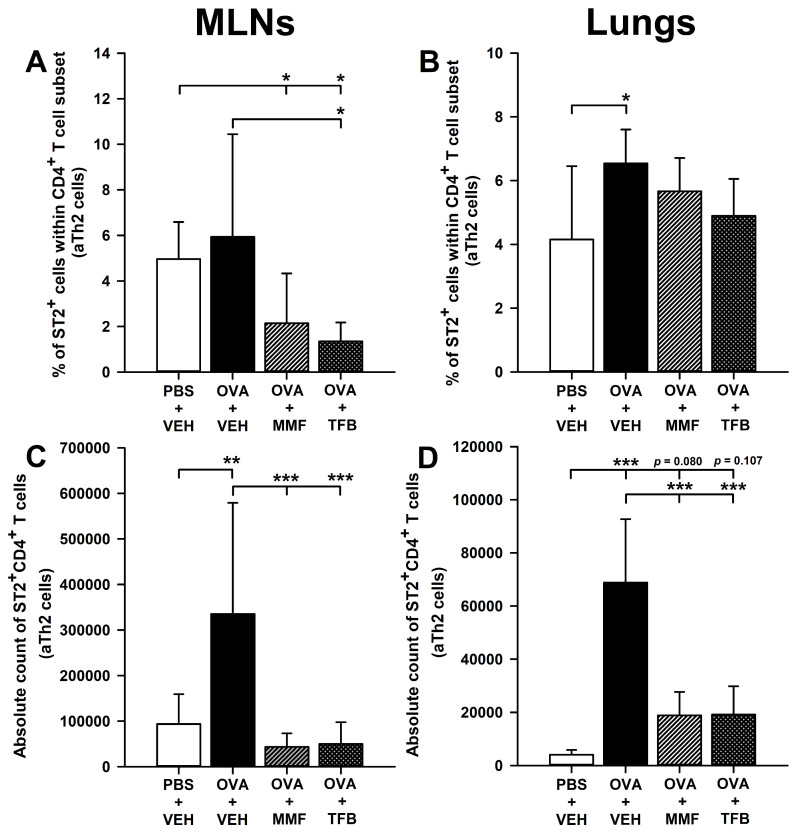
Effect of mycophenolate mofetil (MMF) and tofacitinib (TFB) on the number of activated T helper type 2 (aTh2) cells in the mediastinal lymph nodes (MLNs) and lungs. The relative count (**A**,**B**) is expressed as a percentage of ST2-expressing cells within the CD4^+^ T cell subset. The absolute counts (**C**,**D**) represent the number of ST2^+^CD4^+^ in the whole MLNs and lungs collected from individual mice. Results are expressed as the mean (±S.D.) of two independent experiments with four mice per group (overall *n* = 8 per group, * *p* < 0.05, ** *p* < 0.01, *** *p* < 0.001).

The research showed that immunization with OVA did not affect the percentage of ST2^+^CD4^+^ T cells (i.e., activated Th2 (aTh2) cells) in the MLNs (Figure 5A). The percentage of ST2^+^CD4^+^ T cells in the MLNs of mice from the OVA + MMF and OVA + TFB groups was significantly lower compared to that in the PBS + VEH group; no differences were found between those groups in this respect (Figure 5A). These results strongly suggest that MMF and TFB decreased to a comparable degree the constitutive expression of ST2^+^ on CD4^+^ T cells in the MLNs. The immunization with OVA increased the percentage of ST2^+^CD4^+^ T cells in the lungs (Figure 5B). Interestingly, the percentage of ST2^+^CD4^+^ T cells in the lungs of mice treated with MMF and TFB did not differ significantly from the value of this parameter obtained in both PBS + VEH and OVA + VEH groups (Figure 5B). In view of such results, it seems logical to conclude that both agents reduced to some extent the OVA-induced increase in the percentage of ST2^+^CD4^+^ T cells in the lungs.

The research showed that immunization with OVA led to a significant increase in the absolute number of ST2^+^CD4^+^ T cells in the MLNs and lungs (Figure 5C,D). The absolute number of ST2^+^CD4^+^ T cells was significantly lower in the MLNs and lungs of MMF- and TFB-treated mice compared to that in mice from the OVA + VEH group, and did not differ considerably from the value of this parameter achieved in the PBS + VEH group (Figure 5C,D). Basically, in the context of statistical analysis, these results indicate that the treatment with MMF and TFB prevented the OVA-induced increase in the absolute number of ST2^+^CD4^+^ T cells in both tissues. However, as regards the lungs, the same conclusion may be questionable because the absolute number of ST2^+^CD4^+^ T cells in the lungs of mice from the OVA + MMF and OVA + TFB groups was 4.67 and 4.75 times greater, respectively, than the value of this parameter in the PBS + VEH group. Moreover, although the statistical analysis did not show any significant differences between the treatment groups and the PBS + VEH group, a clear trend toward increasing the absolute number of ST2^+^CD4^+^ T cells in the lungs of mice treated with MMF (*p* = 0.080) and TFB (*p* = 0.107) can be observed (Figure 5D). Therefore, for reasons of scientific caution, it would be more appropriate to conclude that the administration of MMF and TFB almost, but not fully, prevented the OVA-induced increase in the absolute number of ST2^+^CD4^+^ T cells in the lungs.

## 3. Discussion

The results of the histopathological scoring system demonstrated that the administration of MMF and TFB highly attenuated the severity of OVA-induced lung inflammation. These results are in agreement with the results of pulmonary function tests. These tests revealed that the treatment with MMF and TFB ameliorated the development of OVA-induced AHR. In conclusion, the combination of the results of histopathological and functional assessments indicates that the treatment with MMF and TFB highly attenuated, but did not prevent or abolish, OVA-induced AAI. Furthermore, the magnitude of this beneficial effect was comparable between both agents. Admittedly, in light of statistical results, TFB was slightly superior to MMF in pulmonary function tests; however, the differences between them in this respect were small and are unlikely to be clinically significant.

An integral element of these studies was to assess the effect of MMF and TFB on the CD4^+^ T-cell mediated immune response in the MLNs and lungs, i.e., in the inductive and effector sites, respectively, of the immune response underlying the development of allergic asthma. CD4^+^ Teff cells have a central role in the pathogenesis of the disease. Following antigen presentation by dendritic cells to recirculating naive T in the MLNs, allergen-specific CD4^+^ T cells undergo clonal expansion (i.e., proliferate) and differentiate into T helper type 2 (Th2) cells. These cells migrate to the lungs, where they orchestrate pulmonary immune responses and mediate lung inflammation. Th2 cells promote airway inflammation by production of a range of cytokines including IL-4, IL-5, IL-9, IL-13 and IL-25, which together are responsible for the development of hallmark features of asthma [24,25]. Foxp3^+^CD25^+^CD4^+^ regulatory T (Treg) cells have been discovered as another pivotal CD4^+^ T cell subset involved in the pathogenesis of asthma. In this paper, the non-Treg CD4^+^ T cells, i.e., CD4^+^ T cells which did not co-express Foxp3 (the most specific Treg marker, but also a factor which endows them with suppressive properties) and CD25 (α-chain of the IL-2 receptor complex expressed on activated T cells and murine Treg cells), are considered a pool of Teff cells; this is somewhat of a simplification because this subset also includes other regulatory cells, i.e., Tr1 cells.

The absolute counts of CD4^+^ Teff cells and activated Th2 (aTh2) cells were increased in the MLNs and lungs of untreated OVA-immunized mice. The treatment with MMF and TFB prevented these effects in the MLNs. Should these results be analyzed in isolation rather than in the context of the remaining results and related knowledge, it might be concluded that the administration of both agents fully prevented proliferation of allergen-specific CD4^+^ T cells in the MLNs. However, in light of the entirety of results, such a conclusion would be far-fetched and in fact erroneous since the treatment with MMF and TFB very strongly reduced, but did not prevent/abolish, OVA-induced AAI and increases in the absolute number of CD4^+^ Teff cells and aTh2 cells in the lungs. Thus, if the lung tissue of MMF- and TFB-treated mice was infiltrated to a certain degree by CD4^+^ Teff cells and aTh2, it means that these cells were clonally expanded in the lung-draining lymph nodes (i.e., in the MLNs), and recruited into the lungs. It proves that the allergen-specific CD4^+^ Teff cells in the MLNs of mice treated with the tested agents must have proliferated to some extent, but apparently this effect took place before tissue collection and therefore was not captured at the time of the analysis. This assertion is supported by the fact that the absolute number of these CD4^+^ Teff cells in the MLNs of mice treated with MMF and TFB was 1.63 and 1.85 times greater, respectively, than the value of this parameter in the MLNs of non-immunized mice. The results have enabled us to conclude that the impairment of clonal expansion of CD4^+^ Teff cells is a critical event in the mechanism underlying the anti-asthmatic effect of MMF and TFB. A question arises about the first event in this mechanism. With regard to MMF, the answer seems obvious because a substantial mechanism of action of this agent is its antiproliferative effect exerted via IMPDH inhibition. Therefore, it can be stated that MMF directly inhibited proliferation of CD4^+^ T cells in the MLNs, although it cannot be ruled out that this effect was additionally exerted indirectly. As for TFB, it has been demonstrated that this agent exerts an antiproliferative effect on CD4^+^ T cells [26,27]. Taking into account the involvement of the JAK-STAT pathway in lymphocyte proliferation, it should be assumed that TFB indirectly inhibited proliferation of CD4^+^ T cells in the MLNs, i.e., through suppression of pro-proliferative signal transduction in these cells.

ST2 is a receptor for interleukin (IL)-33. The IL-33-ST2 signaling pathway is a pro-inflammatory pathway that plays a significant role in inflammatory diseases by activating the type 2 inflammatory response, producing type 2 cytokines and chemokines [28,29]. Recently, IL-33 has become a hot topic of research because of its role in pulmonary inflammation, and inhibiting the IL-33-ST2 signaling pathway may be a new target for treating allergic diseases, including asthma. Blockade of IL-33 has been shown to inhibit the Th2-type inflammatory pathway, which is the most common pathogenic pathway of asthma [29]. The research showed that immunization with OVA did not affect the frequency of ST2-expressing CD4^+^ T (i.e., aTh2) cells in the MLNs but increased this parameter in the lungs. The results also suggest that both agents to some extent reduced OVA-induced ST2 expression on CD4^+^ T cells in the lungs. What is more, the study demonstrated that MMF and TFB down-regulated the constitutive expression of ST2^+^ on CD4^+^ T cells in the MLNs. These results strongly suggest that suppression of the IL-33/ST2 signaling pathway may constitute an additional mechanism involved in producing the anti-asthmatic effect of both agents.

Treg cells can suppress Th2 and Th17 cell-mediated inflammation and prevent allergic asthma both in asthmatics and in animal models [30,31]. Therefore, generation of inducible Treg (iTreg) cells is considered a new potential approach to the treatment of allergic diseases, such as asthma. In the available literature, there are reports suggesting that the generation of iTreg cells might constitute additional mechanisms underlying the immunosuppressive properties of MMF [32,33,34], although some other studies contradict it [35,36]. As in our previous studies [37,38,39] we found that immunization with OVA induced a robust increase in the abundance of Treg cells in the MLNs and lungs. The treatment with MMF and TFB did not induce an increase in the number of Treg cells, but prevented OVA-induced increase in their number. These results indicate that the anti-asthmatic effect induced by the studied agents is not mediated by the generation of iTreg cells.

A case series report involving 22 patients with steroid-resistant asthma found that the treatment with MMF improved asthma symptoms in half of the patients [40]. Mao et al. [41] found that the treatment with MMF attenuated OVA-induced eosinophil recruitment into the lungs and bronchoalveolar lavage fluid (BALF). In two available studies in which TFB was evaluated in the context of asthma management, it was demonstrated that the agent reduced, but did not abolish, the influx of eosinophils into the BALF [23,42]. To the best of our knowledge, there are no studies evaluating the effect of MMF and TFB on the development of an animal model of AAI by means of the histopathological and functional assessment of the lungs, as was done in this study. Moreover, there are no data on the effect of MMF and JAK inhibitors on CD4^+^ Teff and Treg cell-mediated immune responses in the MLNs and lungs in animal models of lung inflammation. Thus, this is the first report in this respect.

In our previous study, we demonstrated that the treatment with inhaled and systemic glucocorticosteroids prevented OVA-induced AAI in mice [37]. Moreover, in subsequent studies, we found that a blockade of the interaction between the receptor activator of nuclear factor-κB (NF-κB) ligand (RANKL) and its receptor RANK [39], as well as the blockade of NF-κB inhibitor kinase and of NF-κB translocation to the nucleus, also prevented the development of AAI [39,43]. Therefore, in light of these results, it can be stated that the glucocorticosteroid treatment and above-mentioned novel therapeutic strategies have a clear advantage over TFB and MMF with regard to counteracting the development of OVA-induced AAI in mice. However, these new therapeutic strategies have not yet been approved by any regulatory authority, hence, their true effectiveness and safety in clinical settings is unknown. On the contrary, MMF and TFB are approved in many countries and their safety is clinically proven.

## 4. Materials and Methods

### 4.1. Animals

All of the procedures were approved by the Local Ethics Commission (The Local Ethics Commission for Animal Experiments in Olsztyn; Ethical permission No. 54/2023). The experiments were carried out on 6-week-old female Balb/c mice. Mice were bred and maintained under standard laboratory conditions (12/12 h light/dark cycle, controlled temperature (21 ± 2 °C) and humidity (55 ± 5%), and with ad libitum access to autoclaved food and water) in the Animal Facility of the Faculty of Veterinary Medicine, University of Warmia and Mazury in Olsztyn. Mice were euthanized by asphyxiation with CO_2_.

### 4.2. Antigen Immunization, Airway Challenge and Treatment Protocol

**Figure 6:**
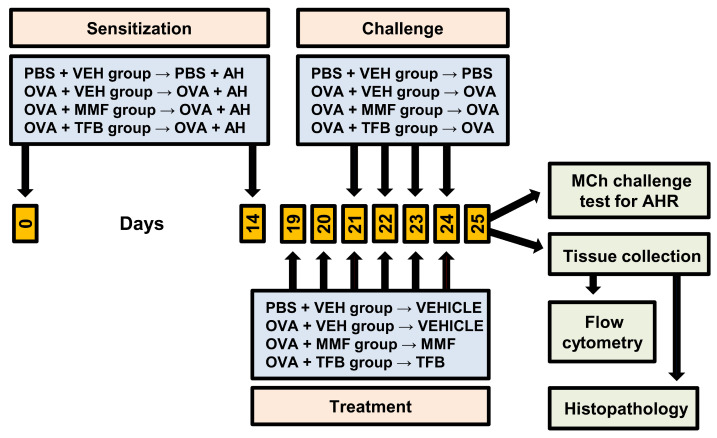
Experimental study design. Mice were divided into four groups, namely PBS + VEH group (PBS-sensitized and -challenged mice treated with vehicle (VEH) used to dissolve the testing agents, i.e., healthy mice/negative control), OVA + VEH group (ovalbumin (OVA)-sensitized and -challenged mice treated with VEH, i.e., mice with an OVA-induced model of allergic asthma/positive control) and OVA + MMF and OVA + TFB groups (i.e., OVA-sensitized and -challenged mice treated with mycophenolate mofetil (MMF) or tofacitinib (TFB), respectively). Mice were sensitized to OVA by two intraperitoneal (i.p.) injections on days 0 and 14 with OVA absorbed on aluminum hydroxide (AH). Subsequently, mice were challenged with aerosolized OVA (OVA + VEH, OVA + MMF and OVA + TFB groups) once daily on days 21–24. Mice in the negative control group (PBS + VEH group) received only AH in PBS (sensitization) or PBS alone (challenge). MMF, TFB and VEH administration was started 48 h prior to the first challenge (i.e., on day 19 after the initial sensitization) and continued for 5 consecutive days. A methacholine (MCh) challenge test (i.e., the measurement of airway hyperresponsiveness (AHR) to MCh) was performed 24 h after the last challenge with OVA or PBS, and subsequently mice were euthanized and the mediastinal lymph nodes and lungs were harvested for histopathology and flow cytometry analysis.

A schematic diagram showing the design of the experiment is presented in Figure 6. The experimental design included the following groups: (I) control groups: (1) the PBS + VEH (vehicle) group (PBS-sensitized and -challenged mice treated with VEH, i.e., healthy mice/negative control) and (2) OVA + VEH group (OVA-sensitized and -challenged mice treated with VEH, i.e., mice with OVA-induced AAI, which is identified as a model of allergic asthma); (II) experimental groups: (3) OVA + MMF (OVA-sensitized and -challenged mice treated with MMF) and (4) OVA + TFB (OVA-sensitized and -challenged mice treated with TFB). Each group included 4 mice and experiments were performed two times (overall n = 8 per group).

Mice were sensitized (under isoflurane anesthesia (Aerrane, Baxter, Deerfield, IL, USA)) on days 0 and 14 via intraperitoneal (i.p.) injection of 20 µg OVA (Grade V) emulsified in 2 mg aluminum hydroxide (AH) (both from Sigma-Aldrich, Schnelldorf, Germany) in a total volume of 200 µL PBS. From day 21 to day 24, the mice were challenged by exposure for 30 min to aerosolized 1% OVA in PBS delivered by an aerogen nebulizer (Buxco FinePointe Whole Body Plethysmography 4-Site System; Data Sciences International, St. Paul, MN, USA). Mice in the negative control group (PBS + VEH group) received only AH in PBS (sensitization) or PBS alone (challenge).

MMF (Sigma-Aldrich) and TFB (Cayman Chemical Co., Ann Arbor, MI, USA) were solubilized in dimethyl sulfoxide (DMSO; referred to as VEH throughout the paper; Sigma-Aldrich) and administered i.p. in doses of 100 mg/kg/day and 20 mg/kg/day, respectively. Mice from the PBS + VEH and OVA + VEH groups were treated with an equivalent volume of DMSO. Administration of VEH, MMF and TFB was started 48 h prior to the first challenge and continued daily for 5 consecutive days; VEH and both studied agents were given 3 h before OVA challenge. MMF [41,44,45] and TFB [46,47,48] doses were chosen according to relevant published reports.

### 4.3. Measurement of Airway Hyperresponsiveness (AHR)

AHR was measured by MCh-induced bronchoconstriction using plethysmography. Penh and PAU, indicators of bronchoconstriction, were measured at baseline and after sequential delivery of increasing concentrations of MCh (Sigma-Aldrich). Briefly, 24 h after the last challenge with OVA or PBS, AHR to MCh was measured in unrestrained, conscious mice by using a 4-chamber, whole-body plethysmograph (Buxco FinePointe Whole Body Plethysmography 4-Site System; Data Sciences International). The mice were placed individually in separate plethysmographic chambers and allowed to acclimatize for 10 min before analysis. Subsequently, mice were first exposed to aerosolized PBS, then challenged with a series of increasing concentrations of aerosolized MCh in PBS (6.25, 12.5, 25 and 50 mg/mL) delivered by an aerogen nebulizer. Each nebulization lasted for 4 min, and then Penh and PAU were measured and averaged for 6 min. Between each dose of PBS and MCh, the airway resistance was allowed to return to the baseline level and stabilize for 5 min before the next MCh dose was administered. Each chamber was equipped with a pneumotachograph (Halcyon™, Buxco, Data Sciences International) to measure the flow and transmit this information to analysis software (FinePointe version 2.9.0, Buxco, Data Sciences International). The patented Halycon^TM^ low noise pneumotachograph reduces disturbances caused by air currents from outside the chambers, which can disrupt or overwhelm the ventilatory airflows within the chamber [49]. Penh and PAU were measured and calculated using FinePointe version 2.9.0. software (Buxco, Data Sciences International), according to the formulas described in the manufacturer’s manual [50].

### 4.4. Lung Histology

Lung sections were prepared and stained as previously described [39]. Airway inflammation was quantified in the peribronchial region of 5 different medium-sized bronchi per slide on the basis of the scoring system previously described [51] and used in similar studies [39]. The results were averaged and totaled for each mouse, and thereafter the mean (±standard deviation (S.D.)) score per group was calculated. Epithelial thickness was measured at 4 sites for 5 different medium-sized bronchi per slide. All measurements were averaged, giving the mean (±S.D.) epithelial thickness per group.

### 4.5. Isolation of Inductive and Effector Site Lymphocytes

#### 4.5.1. MLNs

MLNs were removed and subjected to Dounce homogenization. The resulting cell suspensions were filtered through Nitex fabric (Fairview Fabrics, Hercules, CA, USA), washed with Facs (fluorescence-activated cell-sorting) buffer (FB; Dulbecco’s PBS devoid of Ca^2+^ and Mg^2+^ with 2% (*v*/*v*) heat-inactivated FBS (both from Sigma-Aldrich)), and centrifuged (300× *g* for 5 min. at 5 °C; the same parameters were used for all cell-washing procedures). Cells were re-suspended in FB, counted and stained for flow cytometric analysis.

#### 4.5.2. Lungs

Lungs were minced, subjected to Dounce homogenization, and washed in incomplete medium (RPMI-1640 + 10 mM HEPES buffer + 10 U/mL penicillin/streptomycin; all from Sigma-Aldrich). Subsequently, lung tissues were digested with 50 U/mL (25 mL per sample) collagenase type IV (Sigma-Aldrich) solution containing DNase I (50 U per ml Sigma-Aldrich) in a 50-mL glass flask with a magnetic stir bar. Following rapid agitation during the digestion at 37 °C for 90 min., the resulting cell supernatant was removed and filtered through Nitex fabric, and the cells were washed in complete medium (CM; RPMI-1640 + 10% heat-inactivated fetal bovine serum + 10 mM HEPES (4-(2-hydroxyethyl)-1-piperazineethanesulfonic acid) buffer + 10 mM non-essential amino acids + 10 mM sodium pyruvate + 10 U/mL penicillin/streptomycin; all from Sigma-Aldrich). Isolated cells were re-suspended in a 40% percoll solution (100% percoll solution = 90% of percoll + 10% of 10× Hanks’ Balanced Salt Solution; both from Sigma-Aldrich; dilutions of percoll solution were prepared using CM), and then they were layered over a 60% percoll solution and subjected to gradient centrifugation (400× *g* for 20 min. at 20 °C). Mononuclear cells were removed from the interface layer, washed and then re-suspended in FB and counted.

### 4.6. Flow Cytometry

#### 4.6.1. Extracellular Staining

Cell samples prepared as described above were pre-treated with anti-CD16/CD32 (clone: 2.4G2) FcR blocker for 15 min on ice. Subsequently, the cells were stained for surface antigens with fluorochrome-conjugated monoclonal antibodies (mAbs): PerCP-Cy 5.5 rat anti-mouse CD4 (clone RM4-5, IgG2a, κ), PE-CY7 rat anti-mouse CD25 (clone PC61; IgG1, λ) and PE rat anti-mouse IL-33R/ST2 (clone, U29-93, IgG2a, κ; all from BD Biosciences, San Jose, CA, USA). After 30 min of incubation (on ice and in the dark), the cells were washed in 2 mL of FB.

#### 4.6.2. Intracellular Staining for Foxp3

Cells stained for surface markers were washed, fixed and permeabilized using a mouse Foxp3 buffer set (BD Biosciences) according to the manufacturer’s protocol. Subsequently, the cells were stained with AF-488-conjugated anti-Foxp3 mAb (clone MF23; IgG2b; BD Biosciences). After 45 min of incubation (at RT in the dark), the cells were washed twice with 2 mL of FB and analyzed by flow cytometry.

### 4.7. FACS Acquisition and Analysis

**Figure 7:**
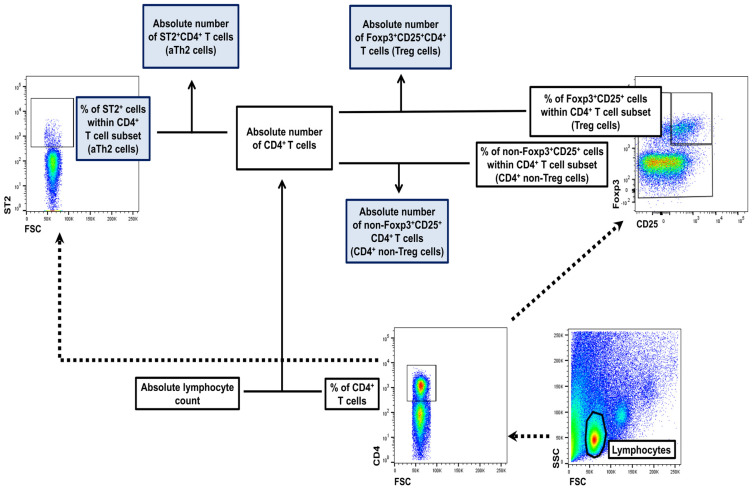
Gating strategy for flow cytometric data analysis and calculation of the absolute cell counts of lymphocyte subsets. Lymphocytes were identified based on forward and side scatter (FSC/SSC) properties, and then gated for the expression of CD4 surface receptor. CD4^+^ T cells were analyzed for the expression/co-expression of CD25 and Foxp3. On this basis, regulatory (Treg; Foxp3^+^CD25^+^CD4^+^) and non-Treg (the remaining CD4^+^ T cells, i.e., non-Foxp3^+^CD25^+^ CD4^+^ T cells ≈ effector T (Teff) cells) cells were distinguished. To identify activated T helper type 2 (aTh2) cells, ST2 (a component of the receptor for IL-33)-expressing cells were gated within the CD4^+^ T cell population. Absolute cell counts of lymphocyte subsets (i.e., number of cells from particular subpopulations per mediastinal lymph nodes or lungs collected from individual mice) were calculated using the dual platform method, as shown above. Solid arrow lines show the direction of the gating while dotted arrow lines represent a way of calculating the absolute count of particular cell subsets.

Flow cytometry analysis was performed using a FACSCelesta cytometer (BD Biosci-ences). The data were acquired by FACSDiva version 9.0 software (BD Biosciences) and analyzed by FlowJo version 10.1 software (Tree Star Inc., Stanford, CA, USA). Absolute cell count was obtained using the dual platform method. Briefly, the total cell count was calculated (using a cell counting chamber) for the whole MLNs and lungs harvested from individual mice. The absolute counts of evaluated cell subsets were determined by recalculating these data by the percentage of particular cell subsets (data from flow cytometry analysis), as illustrated in Figure 7. Thus, the absolute count represented the number of cells from a particular subset in the whole MLNs and lungs collected from individual mice.

### 4.8. Statistical Analyses

Results were expressed as the mean (±S.D.) of two independent experiments with 4 mice per group (overall n = 8 per group). One-way analysis of variance followed by Holm-Sidak multiple comparisons test was used for statistical analysis. Differences were deemed significant when the *p* values were <0.05. SigmaPlot Software Version 12.0 (Systat Software Inc., San Jose, CA, USA) was used for statistical analysis and the plotting of graphs.

## 5. Conclusions

In summary, the treatment with TFB and MMF highly attenuated the development of a mouse model of AAI. The magnitude of the anti-asthmatic effect was comparable between both agents. The impairment of clonal expansion of CD4^+^ Teff cells in the MLNs—which in consequence reduces the infiltration of inflammatory cells into the lung tissue—is a critical event in the mechanism underlying the anti-asthmatic effect of MMF and TFB. Moreover, the suppression of the IL-33/ST2 signaling pathway may constitute an additional mechanism, i.e., acting independently of the inhibition of CD4^+^ T cell proliferation, responsible for producing this effect. In turn, the results indicate that the anti-asthmatic action induced by the studied agents is not mediated by the generation of iTreg cells. Clinical implication of the obtained results: the results suggest that MMF and TFB may exert anti-asthmatic effects, and thus they may be considered therapeutic options for the treatment of allergic asthma cases resistant to conventional/existing treatment.

## Data Availability

The original contributions presented in the study are included in the article, further inquiries can be directed to the corresponding authors.
